# Leg restlessness preceding the onset of motor symptoms of Parkinson disease: A case series of 5 patients

**DOI:** 10.1097/MD.0000000000016892

**Published:** 2019-08-16

**Authors:** Keisuke Suzuki, Hiroaki Fujita, Yuji Watanabe, Takeo Matsubara, Taro Kadowaki, Hirotaka Sakuramoto, Mai Hamaguchi, Narihiro Nozawa, Koichi Hirata

**Affiliations:** Department of Neurology, Dokkyo Medical University, Japan.

**Keywords:** leg motor restlessness, leg restlessness, Parkinson disease, restless legs syndrome

## Abstract

Patients with Parkinson disease (PD) often show restless legs syndrome (RLS), leg motor restlessness (LMR) and other leg restlessness (OLR) related to sensorimotor symptoms.

Here, we describe 5 patients who presented with leg restlessness as an early manifestation of PD.

In case 1, the patient had leg restlessness that was not LMR or RLS and preceded the onset of motor symptoms by 1 year. In case 2, LMR preceded motor symptoms by 2 years. Case 3 had unilateral RLS symptoms on the left side of the body for 33 years. Two and a half years after the spread of RLS symptoms to the right leg with increased frequency of left-sided RLS symptoms, the patient developed PD at the age of 58 years. In cases 4 and 5, RLS symptoms preceded motor symptoms by 3 months and 1 month, respectively. All patients developed Parkinsonism within 3 years (median, 1.0 year; range 0.083–2.5 years) after initial onset or exacerbation of leg restlessness. All patients had frequent leg restlessness symptoms (6–7 days per week). In our series, the preceding leg restlessness was unilateral and confined to the dominant side of the subsequent Parkinsonism, or preceding leg restlessness was bilateral but dominant on the dominant side of the subsequent Parkinsonism.

Clinicians should be aware that late-onset leg restlessness (>50 years of age) including RLS, LMR, and OLR, particularly if frequent and asymmetrical, can be an early nonmotor manifestation of PD.

## 1 Introduction

Restless legs syndrome (RLS) is characterized by the urge to move the legs, usually accompanied by abnormal leg sensation. Because RLS affects sleep as well as daily activities and significantly interferes with quality of life,^[1]^ it is imperative to screen for this condition and manage it. Patients with Parkinson disease (PD), which is characterized by motor and numerous nonmotor signs, show various sensorimotor symptoms including RLS. A meta-analysis showed an increased prevalence of RLS in PD patients compared with healthy controls.^[1]^ RLS and PD have several similarities, including favorable response to dopaminergic drugs, aggravation by the use of dopaminergic antagonists and association with periodic leg movements in sleep; some key differences are that degeneration of nigrostriatal dopaminergic neurons and increased iron in the substantia nigra (SN) are observed in PD, while RLS shows intact nigrostriatal dopaminergic neurons, possible involvement of the diencephalic A11 area and decreased iron content in the SN.^[2]^ These observations suggest that the PD with RLS and idiopathic RLS involve different pathophysiology.

Although there is no clear evidence to suggest that RLS can be a risk factor for PD,^[3,4]^ severe and frequent RLS symptoms (>15 times/month) in men are associated with the development of PD within 4 years,^[5]^ suggesting that RLS is an early manifestation of PD. Additionally, untreated patients with PD are 3 times as likely as healthy controls to have “leg motor restlessness” (LMR), which is characterized by the urge to move the legs but does not fulfill the 4 essential features of RLS.^[6]^ Here, we present 5 PD patients in whom leg restlessness (RLS, LMR, or other leg restlessness (OLR)) preceded the onset of motor symptoms, and we discuss the correlation between leg restlessness and clinical PD symptoms.

## 2 Patients and methods

Five patients with PD who developed leg restlessness prior to the onset of PD and visited the outpatient clinic of the Department of Neurology, Dokkyo Medical University, from 2012 to 2019 were included in this study. All patients provided informed consent for inclusion in the study. Cases 1 and 3 have been reported previously.^[7,8]^ This retrospective observational clinical study including 5 patients did not require the approval of an ethics committee. A diagnosis of PD was made according to the Movement Disorder Society clinical diagnostic criteria for PD.^[9]^ Atypical Parkinsonian syndromes, such as multiple system atrophy, progressive supranuclear palsy, corticobasal degeneration, and secondary Parkinsonism due to medication or bran lesions, were excluded by brain imaging, history taking and clinical examinations. Disease severity and motor symptoms were assessed with the Hoehn and Yahr (HY) staging system and the Japanese version of the Movement Disorder Society-sponsored revision of the Unified Parkinson Disease Rating Scale part III, respectively.^[10]^

## 3 Classification of leg restlessness

RLS was diagnosed when a patient's condition fulfilled the following 4 essential features and was differentiated from RLS mimics:

1. the patient experiences an urge to move the legs, usually accompanied by abnormal leg sensations;
2. the urge to move the legs and the unpleasant sensations begin or worsen during periods of rest or inactivity;
3. the urge to move the legs and the unpleasant sensations are relieved by movement; and
4. the urge to move the legs and the unpleasant sensations occur only in the evening or night or are worse at those times than during the day.^[11]^

LMR was considered to be present when a patient had the urge to move his or her legs but did not fulfill all 3 of the remaining essential features of RLS.^[6]^ When a patient had abnormal, unpleasant sensation in the legs but did not fulfill the criteria of RLS or LMR, he or she was considered to have OLR.

## 4 Other examinations

The Mini-Mental State Examination was administered to assess cognitive function. Cardiac ^123^I-metaiodobenzylguanidine (MIBG) scintigraphy was performed 15 minutes (early phase) and 4 hours (delayed phase) after an injection of 111 MBq of ^123^I-MIBG (Fujifilm RI Pharma Co., Tokyo, Japan). The heart-to-mediastinum (H/M) ratio was then calculated by dividing the count density of the left ventricular region of interest (ROI) by that of the mediastinal ROI. MIBG uptake of <2.2 was defined as abnormal. ^123^I FP-CIT-SPECT imaging (DAT SPECT) was performed 3 hours after an injection of 167 MBq (4.5 mCi). The specific binding ratio (SBR) of the striatum was semiquantitatively determined and analyzed using the QSPECT DAT quantification program (Molecular Imaging Laboratory Inc., Osaka, Japan). An SBR value of <4.5 was considered abnormal.^[12]^ Transcranial sonography (TCS) was performed using a conventional transcranial Doppler sonographic device equipped with a 2.5 MHz transducer to detect SN hyperechogenicity in the midbrain, as previously described.^[13]^ A hyperechogenic area of the SN ≥0.16 cm^2^ on TCS was defined abnormal. A card-type odor identification test (Open Essence (OE), Wako, Japan) was used to evaluate olfactory function^[14]^; an OE score of ≤4 was defined as hyposmia.^[15]^ PD-related sleep disturbances were assessed with the PD Sleep Scale-2, which was designed to assess 15 sleep problems specific to PD.^[16]^ The Epworth Sleepiness Scale was used to evaluate daytime sleepiness.^[17]^

## 5 Case presentation

### 5.1 Case 1

A 73-year-old man with a medical history of prostate cancer presented with bilateral uncomfortable and unpleasant leg sensations lasting for a year. Abnormal sensations, localized in the distal region of the lower legs, predominantly on the right side, occurred mainly during periods of inactivity and at night, but no urge to move was noted. The patient's leg restlessness met the criteria for neither RLS nor LMR and was considered OLR. The patient had no family history or prior medical history of RLS. Neurological examination showed normal cognition and right-side-dominant Parkinsonism. A member of the patient's family had recently noticed that his walking speed had slowed. Brain magnetic resonance imaging findings were normal. DAT SPECT showed bilaterally decreased striatal uptake, and cardiac MIBG uptake was significantly reduced. The OE test showed hyposmia. A detailed history revealed that the patient had had constipation, sleep-talking and nightmares since the age of 50 years. The blood test results, including serum iron, ferritin, and renal function, were unremarkable. A diagnosis of PD preceded by a 1-year history of OLR was made. The patient was treated with levodopa/decarboxylase inhibitor (DCI) at 100 mg/day and rotigotine at 4.5 mg/day, and his motor symptoms and OLR improved.

### 5.2 Case 2

The patient had been treated for obstructive sleep apnea with continuous positive pressure therapy at the sleep medicine center in our hospital since the age of 46 years. The patient started complaining of jerking movements of the left leg at the age of 48 years and left leg cramps and back pain 3 to 4 days per week at the age of 49 years. At the age of 50 years, he noticed daily leg restlessness on the left side; the characteristics included the urge to move, relief during motor activity and worsening at rest, without worsening or occurrence of symptoms during the night or evening. Constipation developed at the age of 51 years, but no episode suggestive of rapid eye movement sleep behavior disorder was reported. Starting at the age of 51 years, the patient noticed tremor in the left hand, and l year later, decreasing stride length and gradually worsening bradykinesia occurred. At 52 years of age, the patient was diagnosed with PD based on left-dominant Parkinsonism and imaging findings. DAT SPECT showed a reduced SBR value in the right striatum, the side contralateral to clinical Parkinsonism. OE tests were normal. Cardiac MIBG uptake was normal at the diagnosis of PD but was reduced in the delayed phase 1 year later. The patient was diagnosed with PD preceded by a 2-year history of LMR. Gradual titration of dopaminergic agents (levodopa/DCI 300 mg/day and selegiline 7.5 mg/day) benefited the patient's Parkinsonism and LMR.

### 5.3 Case 3

The patient had consistently had restlessness in the left arm and leg since the age of 24 years but had not been treated. At the age of 56 years, the frequency of the left-sided RLS symptoms increased, and the abnormal sensation spread to the right leg. At the age of 57 years, the patient presented with insomnia due to bilateral leg restlessness. Her daughter had had RLS since the age of 20 years. Neurological examination showed no abnormalities. Laboratory test results showed normal serum iron and ferritin levels and renal function. The bilateral leg restlessness met the criteria for RLS. Administration of low-dose pramipexole ameliorated the patient's RLS symptoms. However, 1 year later, the patient noticed right shoulder pain, and another 6 months later, she developed a tremor in her right hand. Neurological findings revealed right-dominant Parkinsonism with preserved postural reflexes. The patient was diagnosed with PD according to clinical signs, DAT SPECT and cardiac MIBG 1.5 years after the spread of RLS symptoms from the left limbs to the right leg. DAT SPECT showed a reduced SBR value in the left striatum, which was the side contralateral to the right-sided RLS symptoms that emerged at the age of 56 years and the subsequent right-dominant Parkinsonism.

### 5.4 Case 4

A 70-year-old man with a previous history of depression developed abnormal, burning sensations, and restlessness accompanied by an urge to move in the bilateral legs, predominantly affecting the left side. The symptoms were worse at rest and during evening and were relieved by movement. Three months later, he presented with slowness of movements and gait disturbances. Seven months after the onset of abnormal leg sensations, the patient was hospitalized for further evaluation. The abnormal sensations fulfilled the RLS criteria. A neurological examination revealed left dominant Parkinsonism with postural instability. Brain MRI showed no abnormality. Due to the presence of Parkinsonism combined with the DAT SPECT and cardiac MIBG findings, the patient was diagnosed with PD following RLS. DAT SPECT showed right-dominant impairment of striatal tracer uptake, contralateral to the side of the predominant RLS symptoms. The RLS symptoms resolved after administration of a long-acting dopamine agonist (rotigotine 9 mg/day). Levodopa/DCI (100 mg/day) was started and titrated to 300 mg/day, resulting in significant improvement in Parkinsonism.

### 5.5 Case 5

A 67-year-old man developed restlessness in the left arm, leg and side of the trunk. The patient had an urge to move the affected body parts, and movement temporarily relieved the symptoms. The symptoms predominantly occurred in the evening and at rest. One month later, the patient presented with slowness of the left hand. A neurological examination showed resting tremor of the left hand and left-side dominant Parkinsonism. The patient was diagnosed with early PD and treated with levodopa/DCI and pramipexole. The Parkinsonism and restlessness improved after dopaminergic treatment (levodopa/DCI, 300 mg/day and pramipexole, 1.5 mg/day). DAT SPECT showed reduced right striatal tracer uptake, contralateral to the left-sided RLS symptoms.

## 6 Results

Table 1 shows the characteristics of the 5 patients with leg restlessness included in this study. The presence of the 4 RLS essential features (represented by the URGE acronym) for each patient is described.^[18]^ All patients had frequent leg restlessness symptoms (6–7 days per week). In case 1, OLR preceded Parkinsonism by 1 year; in case 2, LMR preceded Parkinsonism by 2 years; and in cases 4 and 5, RLS preceded Parkinsonism by 3 months and 1 month, respectively. For case 3, although the patient had had RLS restricted to the left side since the age of 24 years, Parkinsonism developed 2.5 years after an increase in the frequency of left-sided RLS symptoms and the spread of RLS symptoms to the right leg. Taken together, our results show that motor symptoms of PD developed within 3 years after the initial onset of LMR and OLR and the spread of RLS symptoms (median, 1.0 year; range, 0.083–2.5 years). In our series, in cases 2, 3, and 5, the preceding leg restlessness was unilateral and confined to the dominant side of subsequent Parkinsonism; however, cases 1 and 4 showed bilateral preceding leg restlessness, and leg symptoms were dominant on the dominant side of the subsequent Parkinsonism.

**Table 1:**
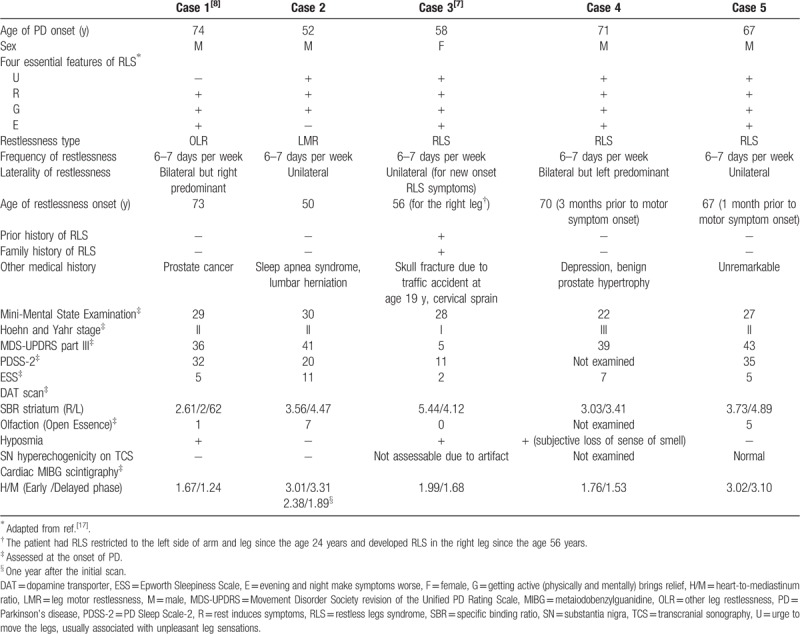
Five PD patients in whom leg restlessness preceded motor symptoms.

## 7 Discussion

Here, we present 5 patients in whom RLS-related symptoms preceded the motor onset of PD within 3 years. Four of the 5 patients (80%) showed unilaterally dominant impairment of striatal tracer uptake on DAT SPECT, contralateral to the side of the predominant RLS symptoms and Parkinsonism. Ondo et al^[19]^ reported that among 87 patients with PD and RLS, PD preceded RLS in 54 (62.1%), RLS preceded PD in 25 (28.7%), and 8 (9.2%) had a concurrent onset. Nomura et al^[20]^ reported that 20 of 165 patients with PD had RLS (12%), and PD preceded RLS in all patients except for 1 patient. In a prospective study of 22,999 men aged 40 to 75 years, 200 subjects developed PD over an 8-year follow-up period. Compared to men without RLS, men who experienced RLS symptoms >15 times/month had a significantly increased risk of PD development (adjusted relative risk, 2.77; 95% CI 1.08–7.11; *P* = .03) within 4 years of follow-up but not within 8 years (adjusted relative risk, 1.47; 95% CI 0.59–3.65; *P* = .41).^[5]^ A large, 2-year, prospective, population-based study including 31,729 (85% men) people showed that individuals who had RLS symptoms 5 to 14 times per month did not show a significant PD risk, but those who had frequent RLS symptoms (15 times or more per month) had a significant PD risk (adjusted odds ratio, 3.09; 95% CI 1.5–6.2; *P* = .003).^[21]^ A study including 16,636 men without PD showed that continuous/recurrent RLS status over 6 years was related to an increased odds ratio of being diagnosed with probable rapid eye movement sleep behavior disorder, known as a significant prodromal marker of PD.^[22]^ These studies suggest that severe and continuous RLS could be an early nonmotor manifestation of PD. However, in prospective studies, 35% to 40% of RLS patients reported continuous RLS symptoms over 2 to 6 years.^[22,23]^ In our series, preceding leg restlessness was unilateral and confined to the dominant side of the subsequent Parkinsonism or was bilateral but dominant on the dominant side of the subsequent Parkinsonism. These findings may support that always unilateral or unilateral dominant leg restlessness could be an early manifestation of PD because laterality of Parkinsonism is commonly observed.^[9]^ In contrast, patients with idiopathic RLS usually show bilateral symptoms. In a study including 44 RLS patients, 28 (64%) showed predominantly bilateral and 26 (59%) showed always bilateral symptoms.^[24]^

An increased prevalence of LMR among untreated patients with PD has been described.^[6]^ In case 3, it is unclear whether the patient's initial RLS symptoms, limited to the left side for 33 years, contributed to the later development of PD. Genome-wide association studies have identified 6 genes and genetic loci associated with RLS, namely, MEIS1, BTBD9, PTPRD, MAP2K5/SKOR1, TOX3, and the intergenic rs6747972 on chromosome 2; none of these markers have been associated with PD.^[25]^ However, it is possible that an unknown gene may be involved in the relationship between RLS and PD.

Dragan et al^[26]^ reported that the onset age of Parkinsonism was older and dyskinesia was less prevalent in the RLS preceding PD group than in the control PD group (without RLS). The investigators suggested that RLS might delay the onset of PD and reduce the severity of PD. Moccia et al^[27]^ found a tendency toward perseveration of dopamine transporter availability in the affected caudate and putamen of PD patients who had suffered from RLS before the onset of PD or who developed RLS and PD at the same time compared with PD patients without restlessness. However, in our series, the onset age of PD was relatively young in 2 patients (case 2, 52 years; case 3, 58 years). In PD patients with RLS, the levels of iron, ferritin, dopamine, and 5-hydroxytryptamine in the cerebrospinal fluid were significantly decreased compared to those of PD patients without RLS.^[28]^ The frequency of depression was higher in PD patients with RLS and LMR than in PD patients without RLS or LMR.^[6]^ The pontine raphe nucleus and coeruleus-subcoeruleus complex, implicated in several nonmotor symptoms, are involved in the premotor stage (Braak PD stage 2).^[29]^ According to these findings, RLS-related symptoms (RLS, LMR, or OLR) could emerge in the early stage of PD, reflecting changes in neurotransmitters in the brain that are not sufficient to cause overt Parkinsonism. In a recent study, 18.2% of 154 early PD patients exhibited restless leg symptoms before the onset of motor symptoms, with a median interval of 70 months from the occurrence of RLS symptoms to the onset of motor symptoms.^[30]^ However, detailed characteristics of leg restlessness was not described.

RLS variants, in which restlessness and unpleasant sensations are limited to or predominantly involve regions other than the legs in patients with PD (lower back and perianal region), have been reported.^[31,32]^ We recently conducted a cross-sectional survey on RLS/LMR and their variants in patients with PD-related disorders; the survey showed that RLS and LMR were observed in 11.5% and 7.7% of untreated patients with PD, respectively.^[33]^ Given that any form of comorbid restlessness was associated with depressive symptoms, insomnia, and autonomic impairment in the study,^[33]^ screening and management of RLS-related symptoms in PD patients is important.

During the premotor phase, olfactory dysfunction can emerge approximately 5 years before the onset of motor symptoms of PD, and hyperechogenicity of the SN on TCS can emerge more than 10 years before the onset of PD motor symptoms.^[34]^ Thus, combined non-invasive evaluation of olfaction and TCS in late-onset RLS patients could be useful for considering whether RLS symptoms are related to an early manifestation of PD. In our series, 60% of individuals showed olfactory dysfunction at PD onset, but none showed SN hyperechogenicity on TCS. SN hyperechogenicity is observed in up to 90% of PD patients.^[35]^ However, compared to Caucasian patients, the prevalence of SN hyperechogenicity may be lower in Asian patients,^[13,36,37]^ and in 15% to 60% of Asian patients, the SN was not assessable due to an insufficient bone window.^[35]^

The study limitations include a descriptive study design and a lack of a healthy control group. Further case-controlled and prospective studies including a large sample of patients are required to confirm our findings.

In this observational study describing 5 PD patients in whom leg restlessness preceded PD onset by a median of 1.0 year, we found that frequent/continuous late-onset leg restlessness (>50 years of age), particularly unilateral involvement, can be an early manifestation of the sensorimotor symptoms of PD. Late-onset RLS with frequent and severe symptoms requires careful observation to determine whether it is an early manifestation of PD.

## Author contributions

**Conceptualization:** Keisuke Suzuki, Hiroaki Fujita, Yuji Watanabe, Takeo Matsubara, Taro Kadowaki, Mai Hamaguchi, Narihiro Nozawa, Koichi Hirata.

**Data curation:** Keisuke Suzuki, Hiroaki Fujita, Takeo Matsubara, Taro Kadowaki, Hirotaka Sakuramoto, Mai Hamaguchi, Narihiro Nozawa.

**Investigation:** Keisuke Suzuki.

**Methodology:** Keisuke Suzuki, Hiroaki Fujita, Yuji Watanabe, Takeo Matsubara, Taro Kadowaki, Hirotaka Sakuramoto, Mai Hamaguchi.

**Supervision:** Koichi Hirata.

**Writing – original draft:** Keisuke Suzuki.

**Writing – review & editing:** Hiroaki Fujita, Yuji Watanabe, Takeo Matsubara, Taro Kadowaki, Hirotaka Sakuramoto, Mai Hamaguchi, Koichi Hirata.
